# Does the model reflect the system? When two-dimensional biomechanics is not ‘good enough’

**DOI:** 10.1098/rsif.2022.0536

**Published:** 2023-01-25

**Authors:** Amanda L. Smith, Julian Davis, Olga Panagiotopoulou, Andrea B. Taylor, Chris Robinson, Carol V. Ward, William H. Kimbel, Zeresenay Alemseged, Callum F. Ross

**Affiliations:** ^1^ Department of Organismal Biology and Anatomy, University of Chicago, 1027 East 57th St, Chicago, IL 60637, USA; ^2^ Department of Anatomy, Pacific Northwest University of Health Sciences, Yakima, WA 90981, USA; ^3^ Department of Engineering, University of Southern Indiana, 8600 University Blvd, Evansville, IN 47712, USA; ^4^ Department of Anatomy & Developmental Biology, Monash Biomedicine Discovery Institute, Faculty of Medicine Nursing and Health Sciences, Monash University, Clayton, Melbourne, Victoria 3800, Australia; ^5^ Department of Basic Science, Touro University, CA 94592, USA; ^6^ Department of Biological Sciences, Bronx Community College, Bronx, NY 10453, USA; ^7^ Doctoral Program in Anthropology, The Graduate Center, City University of New York, New York, NY 10016, USA; ^8^ Department of Pathology & Anatomical Sciences, One Hospital Drive, University of Missouri, Columbia, MO 65212, USA; ^9^ School of Human Evolution and Social Change, Arizona State University, Tempe, AZ 85287-4101, USA

**Keywords:** feeding, strain, biomechanics, mandible, modelling, two-dimensional FEA

## Abstract

Models are mathematical representations of systems, processes or phenomena. In biomechanics, finite-element modelling (FEM) can be a powerful tool, allowing biologists to test form–function relationships *in silico*, replacing or extending results of *in vivo* experimentation. Although modelling simplifications and assumptions are necessary, as a minimum modelling requirement the results of the simplified model must reflect the biomechanics of the modelled system. In cases where the three-dimensional mechanics of a structure are important determinants of its performance, simplified two-dimensional modelling approaches are likely to produce inaccurate results. The vertebrate mandible is one among many three-dimensional anatomical structures routinely modelled using two-dimensional FE analysis. We thus compare the stress regimes of our published three-dimensional model of the chimpanzee mandible with a published two-dimensional model of the chimpanzee mandible and identify several fundamental differences. We then present a series of two-dimensional and three-dimensional FE modelling experiments that demonstrate how three key modelling parameters, (i) dimensionality, (ii) symmetric geometry, and (iii) constraints, affect deformation and strain regimes of the models. Our results confirm that, in the case of the primate mandible (at least), two-dimensional FEM fails to meet this minimum modelling requirement and should not be used to draw functional, ecological or evolutionary conclusions.

## 1. Introduction

Over the last few decades, classical biomechanical modelling of vertebrate mandibles, such as free body diagrams [1] and analyses of cross-sectional geometry [2–4] have been supplemented by increasingly sophisticated finite-element analyses (FEA). Finite-element analysis is a powerful engineering tool for evaluating the mechanical performance of complex biological systems enabling testing of specific mechanical hypotheses about form–function relationships in the context of precise inferences and assumptions [5–7]. Finite-element analysis is the mathematical solution of the differential equations generated from the data of interest [5]. The processual technique, including several stages from model creation through model solution, post-processing and validation [6,7] is referred to as finite-element modelling (FEM). Using FEM, complicated biological structures are partitioned into many simple geometric elements so mechanical metrics such as stresses and strains (physical responses to loads) can be calculated and relationships between shape and function can be explored. Early works discretized the geometry of interest in two dimensions [8–10] but the advancement of and accessibility to computing power and high-quality medical imaging has made it possible to incorporate more accurate three-dimensional external and internal anatomical detail into FE models. Three-dimensional FEM and analysis allow researchers to test mechanical hypotheses by incorporating realistic geometric (using computerized tomography (CT) scans), hypothesized (reconstructed) or idealized form while controlling for or testing the effects of complex variables such as material properties and boundary conditions (external forces and constraints) [11–52]. Still, three-dimensional FEMs can be time consuming to create and computationally expensive to solve, leading some authors to use simplified two-dimensional models in their analyses [42,47,53–57]. Because two-dimensional models require more assumptions about the geometry, forces and constraints than three-dimensional models, we argue that they require more *a priori* knowledge and careful simulation of the mechanics of that system. In order to assess whether the assumptions are reasonable and two-dimensional simplification is justified, a model must exhibit realistic biomechanical performance.

Problems that arise from a two-dimensional modelling approach can be cogently demonstrated in primates, because extensive experimental data on primate feeding biomechanics allow for direct and indirect model validation [26,58–65]. It has long been recognized that three-dimensional mechanics are important for the study of primate mandibles, because the mandible is three-dimensional, and external forces acting on the mandible (the loading regime) include force vectors with significant components in all three dimensions [62,63,66,67]. As a result, primate mandibles are subjected to significant torques about axes oriented anteroposteriorly—i.e. long axis twisting—and superoinferiorly—i.e. wishboning. Considering that experimental evidence confirms significant external forces acting about all three axes [26,68], they all must be considered when analysing the mechanics [26,45]. This applies not only to free-body analyses, estimating external reaction forces acting at bite points and jaw joints, but also to FEM analyses of internal stresses and strains [2,3,69–72]. Furthermore, without all three dimensions, planar two-dimensional models cannot provide insight into relationships between cross-sectional geometry and the mechanical performance of the mandible.

In addition to primates [56], two-dimensional models of early mammal [55], early tetrapod [54], capitosaur [53], crocodilian [47], cingulate [73] and ungulate [42] mandibles have recently been deployed. In some cases, the two-dimensional models might be justified. If the research question is limited to a planar analysis of a structure that is long in one direction and relatively flat in another, then a parasagittal plane model may be sufficient. But the principal loading, deformation and strain regimes must remain in the modelled plane. If results of the analyses are used to make conclusions in three dimensions, they should be interpreted with caution and validated using experimental data. For extinct taxa, or those for which *in vivo* data cannot be collected, direct model validation—comparison of FEA results with *in vivo* experimental results—is impossible, but model credibility can still be established through indirect validation—comparison of FEA results with previously published *in silico* results from closely related taxa [45]. Simplified two-dimensional models may be useful insofar as they allow researchers to generate mechanical hypotheses that can be tested in future work using more detailed three-dimensional models [42,47,57]. For example, ungulate mandibles are long anteroposteriorly and thin mediolaterally, but they experience significant internal and external transverse forces that cannot accurately be accounted for with sagittal two-dimensional models [74,75]. Researchers responsibly point out that two-dimensional ungulate mandible models are a ‘first step’ [42], but we are unaware of follow-up studies that have successfully established the credibility of two-dimensional models of the jaw.

Our principal criticisms of two-dimensional models of the jaw are that (i) the models are flat, and (ii) they only represent half of the mandible (hemi-mandible). Flat models do not accurately represent mandibular three-dimensional geometry and can only provide data in a single plane. Moreover, hemi-mandible models have not accounted for external forces transferred across the symphysis. As a result, previously published two-dimensional models show no strain anterior to the bite point, suggesting they are improperly constrained and loaded [56].

To illustrate these problems, we compared our previously published three-dimensional model [45] with a previously published two-dimensional model [56] of the chimpanzee mandible. Differences in stress regime led us to predict that three key modelling parameters affect deformation and strain regime: (i) the dimensionality, (ii) symmetry, and (iii) the boundary conditions. Next, we conducted a series of *in silico* experiments in which we systematically simplified our three-dimensional model to isolate and test these three parameters. In doing so, we aimed to test assumptions of two-dimensional model simplifications. We reasoned that if we could accurately account for symmetry and boundary conditions associated with the three-dimensional mandible using our simplified models, we may be able to demonstrate comparable biomechanical performance (stress and strain) of the two-dimensional and three-dimensional models. This would indicate that the simplifications may be good enough to understand the overall biomechanics of the system.

Instead, our results show that the omission of all data in the third dimension severely limits the accuracy and, hence, utility of recently published two-dimensional FEMs of primate mandibles [56]. When compared with directly [26] and indirectly [45] experimentally validated three-dimensional FEMs, strain patterns reported for the simplified models do not resemble the deformation and strain regimes that have been shown to occur in the primate mandible *in vivo*. As a best practice, conclusions regarding the function, ecology and evolutionary history that are based on inaccurate models should be interpreted with scepticism [56].

Although here we use primates to demonstrate the problems with two-dimensional FEA, our results and conclusions are likely to extend to mandibles of other taxa and to other structures with significant three-dimensional geometry.

## 2. Material and methods

### 2.1. FEM construction

We constructed our three-dimensional model from µCT scans of a female chimpanzee mandible using methods outlined previously [45]. Briefly, we used Mimics v. 20 (Materialise NV, Leuven, Belgium) to segment out the mandible and create three-dimensional surface meshes of the anatomical tissues of interest (cortical bone, teeth and trabecular bone), Geomagic Studio v. 12 (3D Systems, Research Triangle Park) to repair minor geometric errors in the surface models, and 3-Matic v. 12 (Materialise) to create volumetric meshes. Meshed volumes were imported to FEA software Strand7 (Strand7 Pty Ltd, Sydney) to apply loads and constraints and to perform linear static FEA. The complete three-dimensional model was composed of 2 587 382 four-noded tetrahedral brick elements.

To create the three-dimensional hemi-mandible, we divided the complete three-dimensional model into left and right halves by creating brick entity sets that could be hidden or removed. All elements to the right of the symphyseal midline were then deleted.

**Figure 1.**
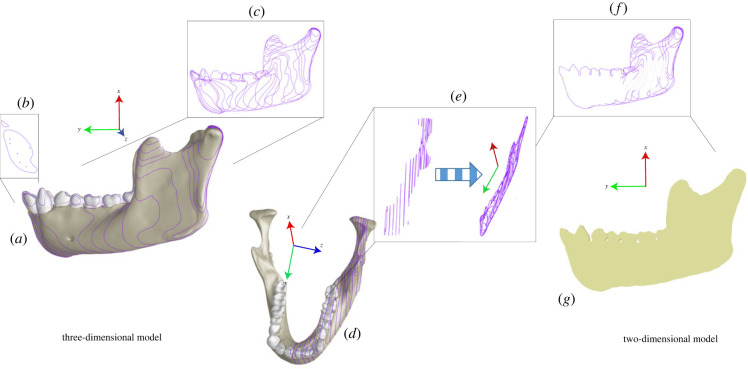
Schematic overview of the three-dimensional to two-dimensional modelling process. (*a*) Left lateral view of complete three-dimensional model, with purple isolines corresponding to cuts made in the sagittal (XY) plane to capture topography of hemi-mandible. (*b*) Shows the outline of the cut at the symphysis/ mid-sagittal plane. (*c*) Bricks deleted, leaving only isolines. (*d*) Superior/oblique view showing isolines. The bricks are then deleted and (*e*) the isolines are collapsed/to the same Z-origin plane. (*f*) Internal elements are then cleaned, connected as a single polygon, and used to create (*g*) a face that is meshed as plate elements.

Previous workers have used photographic projection to render the three-dimensional geometry of the mandible in a single plane rather than selecting a real parasagittal plane passing through the mandible. This results in a flat (cortical bone) model that approximates the shape of the external mandibular (labial/buccal) surface. We chose to create our two-dimensional models directly from our three-dimensional models in Strand7 using a merged multi-slice technique (figure 1). To do this, a new user coordinate system (UCS) was created in the mid-sagittal (XY symmetry) plane and used to cut the brick elements through which it passed (figure 1*a,b*). The UCS was then offset laterally and used to create additional cuts (16 in total) at intervals that captured the anatomical ‘outline’ of the corpus, ramus and teeth in lateral view (figure 1*c*). Beam elements were created on the free edges of each cut to create isolines corresponding to the topographic anatomy of the left side of the mandible (figure 1*d*). Next, all isolines were projected to a single z-origin plane and all internal beam elements were deleted, leaving a single two-dimensional outline of the mandible (figure 1*e*). The elements of the outline were cleaned (to connect as a single polygon), used to create a face and meshed using plate elements (figure 1*f*,*g*).

Plate elements were assigned a uniform thickness for post-processing corresponding to the average thickness (15.34 mm) of the mandibular corpus measured at three points (the back of the M3, mid-toothrow and mid-P3) following techniques described in a previous two-dimensional analysis of the primate mandible [56]. The final two-dimensional plate model was meshed Strand7 and contained 73 534 plate elements connected at 222 023 quad 8 nodes.

### 2.2. Material properties

Material properties of great ape craniofacial cortical bone were applied and considered isotropic and heterogeneous as in Smith *et al*. [45,76,77]. In the three-dimensional models, trabecular bone was considered a volume of bulk tissue [78] and enamel was modelled as ‘caps’ on the surface of the teeth [79]. Although strictly speaking, a planar two-dimensional model of the mandible should be a composite—any parasagittal plane would pass through the ramus/corpus somewhere and should, therefore, consist of an outer cortex (cortical bone and dental enamel) encircling deep trabecular bone. Without depth, two-dimensional planar models cannot include embedded volumes, and previous studies have assigned only the material properties of cortical bone. This is neither realistic geometry nor material properties, but we assigned our two-dimensional model the material properties of only cortical bone to replicate the conditions of previous work, We expect this will lead to a model that is stiffer than our three-dimensional mandible.

### 2.3. Muscle modelling

Forces corresponding to the anterior and posterior temporalis, superficial and deep masseters, and medial pterygoids were applied to both three-dimensional and two-dimensional models. For three-dimensional models, muscle forces were derived from *Pan* muscle physiologic cross-sectional areas, scaled by *Macaca* electromyography (EMG), and directed towards the centroid of cranial insertion areas using Boneload (table 1, electronic supplementary material, figure S1 and [45]. For the two-dimensional models, force magnitudes were calculated (omitting z-components) using the same data and applied as nodal forces distributed across the surfaces of each model corresponding to the regions of muscle attachment. Because only two dimensions were available, medial pterygoid and masseters were applied to the same surface (i.e. they overlapped).

**Table 1.** Muscle forces applied to models.

	force applied to FEMs (N)		
	side	three-dimensional model^a^	two-dimensional model
anterior temporalis	left	111.93	111.93
	right	154.48	0
posterior temporalis	left	55.10	55.10
	right	128.40	0
superficial masseter	left	302.10	302.10
	right	154.34	0
deep masseter	left	9.25	9.25
	right	47.18	0
medial pterygold	left	86.53	86.53
	right	31.46	0

^a^As per Smith *et al*. [45].

### 2.4. Constraints

Boundary conditions were varied across experiments (electronic supplementary material, figure S3). In our three-dimensional, complete mandible models, we applied boundary conditions at the mandibular condyles and bite point (each a single node) to minimally constrain movement and prevent rigid body motion while simulating joint and bite reaction forces [18,44,45,80]. The working-side (arbitrarily chosen as the left side) condyle was fixed against all translations and rotations while the balancing-side (right) condyle was constrained against displacement in the superoinferior (SI) and anteroposterior (AP) directions. The occlusal surface of M1 was constrained against all displacements. Our two-dimensional model was constrained in a similar manner as the working-side of our three-dimensional model (a single node at the condyle and the bite point).

We applied symmetry constraints at the symphysis of our hemi-mandible model, forcing the symphysis to remain in the mid-sagittal plane—no motion occurs perpendicular to that plane. This may result in unrealistic reaction forces at the plane of symmetry and force symmetric displacement (simulating bilateral biting). Because of this, one must be especially cautious when interpreting results for antisymmetric—unilateral—biting conditions. In addition, the boundary conditions at the condyle may over-restrain the symmetric model when compared with a real biting condition, not allowing for realistic motion perpendicular to the sagittal plane. Each of these unrealistic constraints might result in unrealistic joint and bite reaction forces.

### 2.5. Metrics

We compared the results of our models using both qualitative (colourmaps and deformation plots) and quantitative (nodal stresses and strains) metrics (electronic supplementary material, figure S2: key to loading and deformation regimes). This enabled us to validate our modelling methods while still allowing for qualitative comparison of the mechanical performance of our models to published two-dimensional models. Although Marcé-Nogué *et al*. [56] report von Mises stresses, we prefer to report von Mises, shear and axial strains to facilitate comparison with experimental strain studies. (Note that stress and strain are linearly related to each other in this model.) For this reason, we report stresses when comparing our models with the published two-dimensional model, but strains when comparing our own models.

## 3. Results

### 3.1. Comparison of published three-dimensional and two-dimensional models

**Figure 2.**
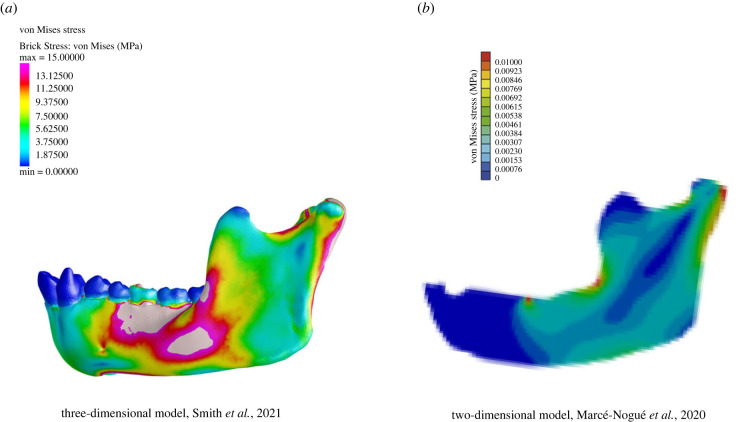
Comparison of stress regimes in the lateral working-side (left) corpus and ramus of three-dimensional [45] and two-dimensional *Pan* models [56]. Stress scale in the three-dimensional model is 0–15 MPa and 0–0.01 MPa in the two-dimensional model. (*a*) von Mises stress regime of three-dimensional model during simulated molar bite, and (*b*) von Mises stress regime of two-dimensional model during simulated molar bite.

In our three-dimensional model, von Mises stresses are highest inferior to the bite point (38 MPa), extending anteriorly and posteriorly along the alveolar process of the corpus and inferior to the external oblique line (figure 2*a*). Strains are also elevated along the base of the mandible (peak of 19 MPa), the mandibular notch (peak of 13 MPa) and the posterior ramus (peak of 12–17 MPa inferior to the condylar head). Relative to the corpus, strains are lower across the ramus. Between the apex of the coronoid process and the mandibular angle, strain magnitudes range from 1 to 10 MPa, with peaks mid-ramus. Conversely, Marcé-Nogué's two-dimensional model (figure 2*b*) shows a distinctive strip of very low von Mises (0–0.000307 MPa) stress in the ramus extending anteriorly and inferiorly from the condylar process towards the corpus, a region that ranges from 1 to 14 MPa in our three-dimensional model. In Marcé-Nogué's model, stresses appear to peak at the bite point, the junction of the corpus and ramus, and the posterior aspect of the condylar head and ramus. No stresses appear anterior to the bite point in the two-dimensional model but range from 1 to 14 MPa in the three-dimensional model.

Differences in modelling technique are undoubtedly responsible for the notable differences in strain regime between these models and predict that modification of three key variables accounts for the differences in results. To better understand these effects, we modified (i) the dimensionality, (ii) the symmetric geometry and loading regime, and (iii) the constraints of our three-dimensional model.

### 3.2. Modelling experiments

#### 3.2.1. Experiment 1: loss of third dimension (three-dimensional versus our two-dimensional model)

**Figure 3.**
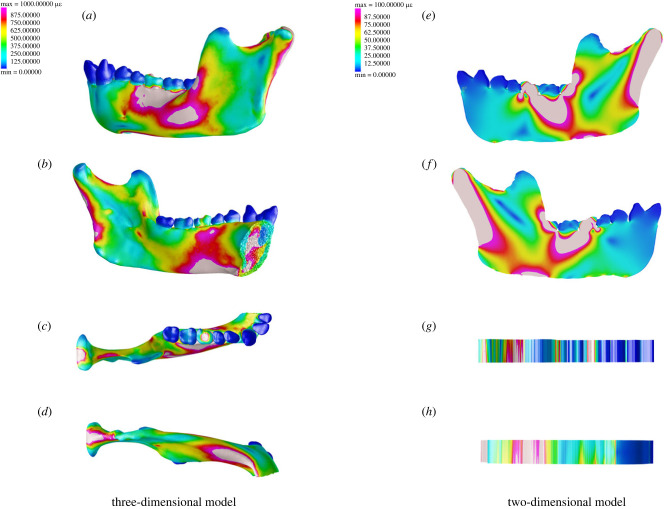
Comparison of strain regime in the working-side (left) corpus, ramus and symphysis of our three-dimensional and two-dimensional models. Strain scale in the three-dimensional model is 0–1000 µ*ε* and is 0–100 µ*ε* in the two-dimensional model. (*a*) Von Mises strain regime in the labial/buccal three-dimensional model during simulated molar bite, (*b*) lingual aspect, (*c*) superior aspect, (*d*) inferior aspect and (*e*) von Mises strain regime in the lateral aspect of two-dimensional model during simulated molar bite, (*f*) medial aspect, (*g*) superior ‘aspect’ *, (*h*) inferior ‘aspect’ *. *Please note that superior and inferior ‘aspects’ of the two-dimensional model are a consequence of visualizing the two-dimensional model in three-dimensional modelling environment. Meaningful data cannot be collected from these edges.

Because the two-dimensional model only represents sagittal geometry, it does not have buccal and lingual surfaces. Strains are identical on both ‘sides’ of a two-dimensional model and the colourmap of its ‘medial’ aspect (figure 3*f*) is simply a consequence of rendering the two-dimensional model in three-dimensional space. Moreover, no meaningful data can be collected from either its superior or inferior aspects (figure 3*g*,*h*; compared with three-dimensional model, figure 3*c*,*d*). Thus, we limit our consideration of the two-dimensional model to figure 3*e*. Here, von Mises strains peak (white on colourmap) inferior to the condyle and are highest along the posterior edge of the ramus (approx. 200 µ*ε*). Strains in the biting tooth peak at approximately 600 µ*ε* with elevated strains inferior to the bite point, extending posteriorly toward the ramus and posteroinferiorly towards the base of the mandible below the coronoid process. Strains are lower anterior to the bite point, but small areas of elevated strains are present (20–100 µ*ε*).

In our three-dimensional model (figure 3*a*,*b*), strains are elevated at the alveolar process between the M1 and P3 (approx. 1000 µ*ε*), with peak strains (up to approx. 1600 µ*ε*), but our three-dimensional model shows clear differences in buccal and lingual strain regimes (figure 3*a*,*b*,*e*,*f*)—data that are lost in the two-dimensional model. On the lingual aspect, strains are highest inferiorly and extend along the basal border inferior to M1/M2 anteriorly toward the symphysis (figure 3*b*,*d*) while on the buccal aspect, elevated strains are concentrated near the toothrow and external oblique line (figure 3*a*,*c*).

Strains are also elevated (figure 3*b*) along the posterior aspect of the ramus inferior to the condylar head (1100 µ*ε*), mandibular notch (975 µ*ε*), the pharyngeal (950 µ*ε*), endocoronoid (850 µ*ε*) and endocondylar crests (650 µ*ε*), and anteroinferiorly from the mandibular foramen, inferior to the alveolar prominence toward the peak strains at the anterior mandibular base (450–850 µ*ε*).

Elevated strains along the posterior ramus and mandibular notch are present for both two-dimensional and three-dimensional models, but the two-dimensional model also has elevated strains at the base of the mandible, inferior to the coronoid process and the area between the anterior ramus and the M1. A region of very low strain extends anteroinferiorly from the mandibular notch towards the anterior ramus of the two-dimensional model (figure 3*e*). The lingual aspect of our three-dimensional model experiences low strains in the area above and behind the mandibular foramen but displays elevated strains in the endocondylar ridge, immediately above. In fact, strain patterns in our two-dimensional model appear reversed relative to our three-dimensional model in regions corresponding to crests and fossae. In our three-dimensional model, strains are high along the endocondylar ridge and endocoronoid crest but low at the masseteric fossa. In our two-dimensional model, strains are low in the area of the endocondylar ridge and endocoronoid crest and high at the masseteric fossa. These features are defined precisely by their variation in topography in the transverse plane, and thus are obliterated in two-dimensional analyses.

**Figure 4.**
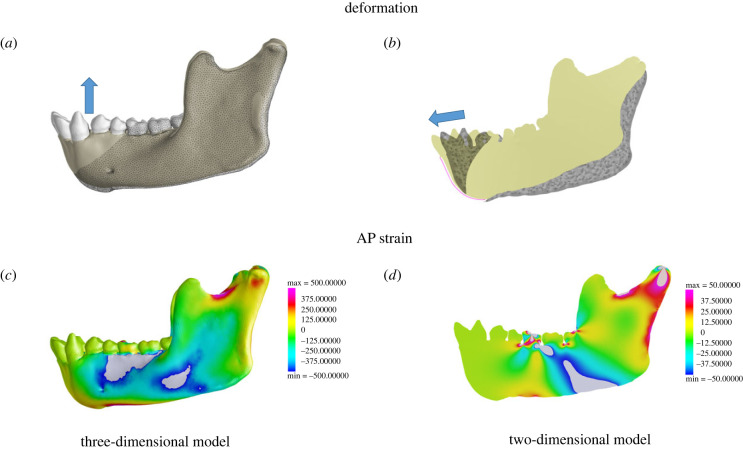
Comparison of deformation and AP strain regime (sagittal bending) in our three-dimensional and two-dimensional models in lateral view. (*a*) Deformation of the three-dimensional model during simulated molar bite and (*b*) deformation of the two-dimensional model during simulated molar bite. The original (undeformed) model is illustrated by the wireframe. The deformation is magnified by 10× for visualization (solid) and blue arrows represent primary direction of deformation. Colourmaps show anteroposterior (AP) strains on the lateral aspect of the corpus and ramus in the (*c*) three-dimensional and (*d*) two-dimensional models. Note difference in scales.

Previous work [26,45,58,61] has shown that the working-side corpus and ramus experience positive sagittal bending, lateral transverse bending and negative twisting about an AP axis, but two-dimensional FEA can only model loading regimes in the sagittal plane (electronic supplementary material, figure S4). Thus, if two-dimensional FEA has any utility in understanding how the primate mandible functions, it should at least accurately reproduce strains associated with the sagittal bending regime. Figure 4 shows the deformation and strain regime for our two-dimensional (figure 4*a*,*c*) and three-dimensional models (figure 4*b*,*d*) in lateral view. In the three-dimensional model, negative AP strains at the alveolar process of the labial corpus below the premolars and molars and positive AP strains at the mandibular base indicate positive sagittal bending as the mandible is deformed downwards by reaction force at the bite point. By contrast, the two-dimensional model experiences negative sagittal bending as the entire corpus and ramus deflect upwards between the inferiorly directed bite reaction force and superiorly directed muscle forces. Flexure occurs about the M1 bite point causing compressive strains that extend to the mandibular base (electronic supplementary material, Animation S1).

Although we do not directly compare nodal strain magnitudes between the three-dimensional and two-dimensional models, peak strains differ by up to an order of magnitude. This is likely to be a consequence of increased stiffness in the two-dimensional model with only cortical and without trabecular bone.

The two-dimensional model reproduces neither the strain nor deformation regime of the three-dimensional condition.

#### 3.2.2. Experiment 2: hemi-mandible (three dimensional, but just half)

Another difference between three-dimensional and two-dimensional models is the presence or absence of symmetric geometry, i.e. another hemi-mandible coupled at the symphysis. Thus, to test whether differences in deformation and strain regime can be explained by omission of half of the mandible, we created a hemi-mandible model by duplicating our three-dimensional model and deleting all brick elements to the right of the mid-sagittal plane (figure 1 and electronic supplementary material, figure S3). For experiment 2 (2a; electronic supplementary material, figure S3), we applied symmetric boundary conditions to newly exposed nodes along the mid-symphyseal surface. If differences between our complete three-dimensional and our two-dimensional models are due only to the ‘flattening’ of the mandibular morphology, we would expect similar mechanical behaviour for our three-dimensional complete and hemi-mandible models.

**Figure 5.**
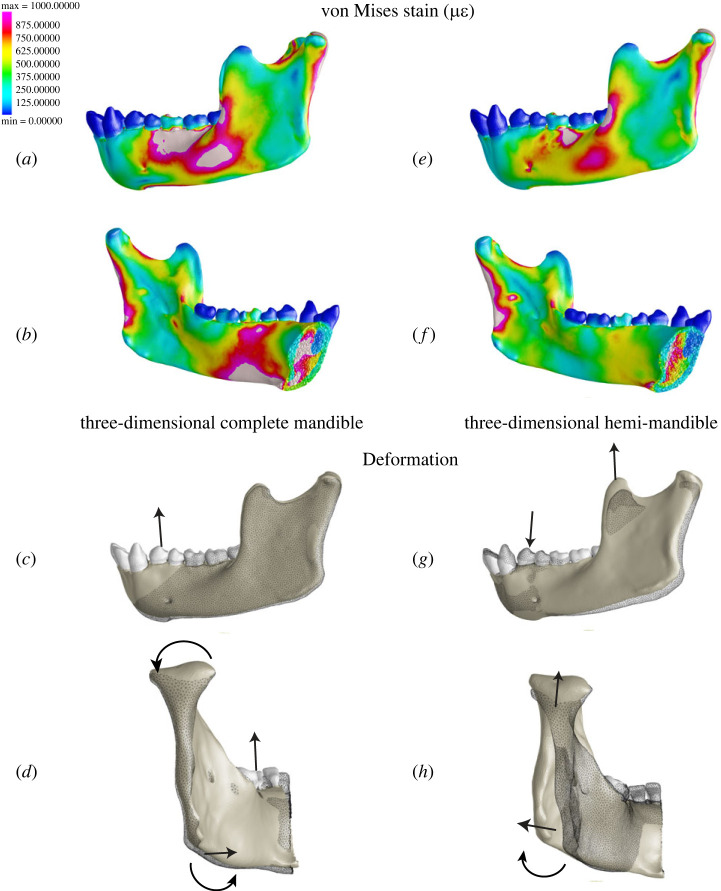
Comparison of strain and deformation regimes in three-dimensional complete and hemi-mandible models. (*a*) Complete model in lateral and medial views, (*b*) hemi-mandible in lateral and medial views, (*c*) complete model in lateral and posterior views and (*d*) hemi-mandible in lateral and posterior views. Colourmaps show von Mises strain (strain scale is 0–1000 µ*ε*). The original (undeformed) model is illustrated by the wireframe. The deformation is magnified by 10x for visualization (solid). Curved arrows represent torsion and straight arrows represent bending (sagittal and transverse).

Although the hemi-mandible model approximates the von Mises strain distribution in parts of our three-dimensional model at the buccal corpus and posterior edge of the ramus (figure 5*a*,*e*), strain differences at the lingual corpus and ramus (figure 5*b*,*f*) indicate critical differences in deformation regimes (figure 5*c*,*d*,*g*,*h*). A lateral view illustrates elevation of the anterior toothrow in our complete model (figure 5*c*). By contrast, our hemi-mandible is depressed anterior to the bite point while the posterior corpus and ramus are elevated (figure 5*g*). Differences in transverse bending and AP twisting are illustrated in posterior view (figure 5*d*,*h*). The corpus and ramus of our complete model show negative AP twisting and medial transverse bending respectively (inversion of angle, eversion of toothrow) while the hemi-mandible shows the opposite—positive AP twisting and lateral transverse bending (eversion of angle, inversion of toothrow).

**Figure 6.**
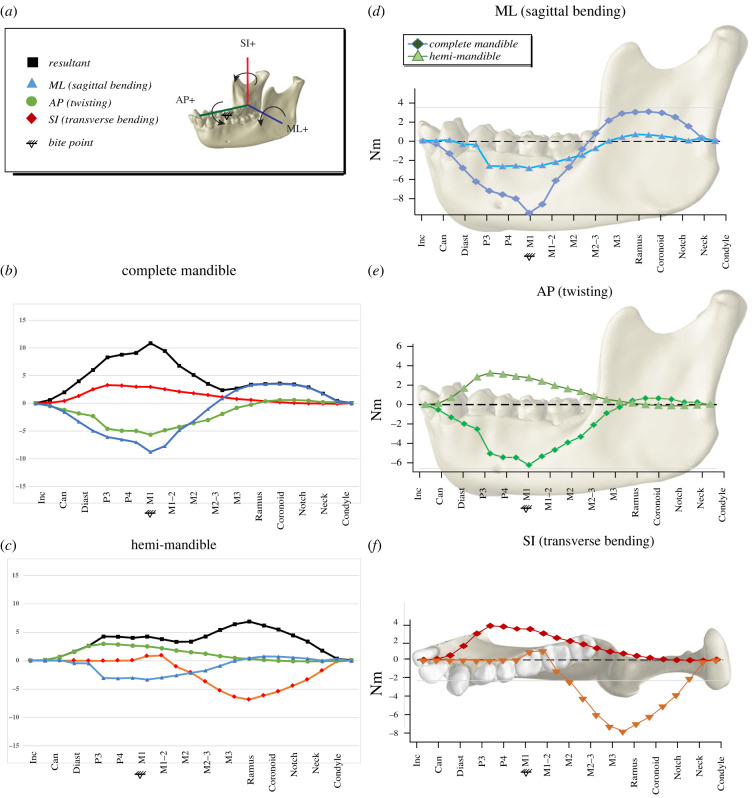
Comparison of moments (in Nm) acting about axes through coronal sections in three-dimensional complete and hemi-mandible models. Moments about ML axes are sagittal bending moments (blue, triangle); moments about AP axes are twisting moments (green, circle); moments about SI axes are transverse bending moments (red, diamond). Moments in the (*a*) complete three-dimensional model and (*c*) three-dimensional hemi-mandible and shown. (*b*) Difference between moments is shown with shading. Images on the right show moments mapped onto the mandible for the complete (diamond) and hemi-mandible (triangle) in (*d*) ML, sagittal bending; (*e*) AP, twisting; (*f*) SI, transverse bending.

Differences in deformation illustrated in figure 5 can be explained by differences in moments between the models—illustrated in figure 6. Differences in moments are driven by differences in the relative proportion and direction of reaction force components between models. In our three-dimensional model, overall torque magnitude tends to be greater at or anterior to the bite point than posterior to the bite point, with the resultant peaking at the bite point (figure 6*a*). Negative sagittal bending moments are the largest acting on the complete mandible, and they peak at the coronal plane through M1, producing positive sagittal bending deformation. High sagittal bending moments of the corpus can be explained by high absolute magnitude (−119 N, −341 N) and relative proportion (82%) of the vertical components of working- and balancing-side reaction forces. Horizontal components are relatively low, accounting for only 2% of reaction force.

By contrast, no balancing-side forces exist for the hemi-mandible, and symmetry constraints at the symphysis (anterior) prevent out-of-plane (horizontal) translation. Torques are small (or zero) anterior to the bite point and the resultant peaks more posteriorly (figure 6*c*). The largest moments acting on the hemi-mandible are transverse bending moments, which peak in the vicinity of the masticatory muscle insertions to produce lateral transverse bending deformation of the ramus. The omission of balancing-side forces (and addition of symphyseal symmetry constraint) results in a fourfold increase in the relative proportion of horizontal reaction force components (73% vertical, 9% horizontal). In the absence of any synergistic balancing-side forces, most deformation of the isolated working-side mandible occurs at sites of muscle force application (figure 5*d*). This fundamental shift in loading regime explains the main difference in deformation regime between the two models.

**Figure 7.**
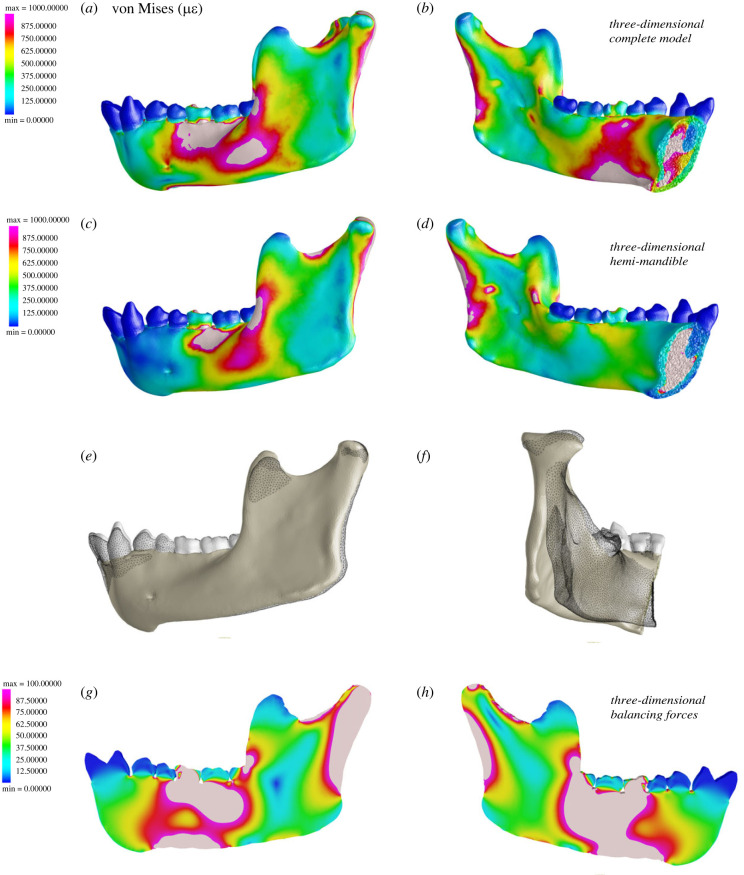
Deformation and strain regime in three-dimensional and two-dimensional hemi-mandible when simulating balancing-side forces. Top images show (*a*) von Mises strain in the lateral aspect of the complete model during simulated molar bite, and (*b*) in medial view. Scale is 0–1000 µ*ε*. Middle images show the strain (*c*) in lateral and (*d*) medial view, and deformation in (*e*) lateral and (*f*) posterior views when forces are applied at the symphysis to simulate balancing-side forces transmitted across the symphysis. Bottom images show the strain in the (*g*) lateral and (*h*) medial aspects of our two-dimensional model. Scale is 0–100 µ*ε*.

It is in theory possible to simulate the transmission of these counterbalancing moments by applying appropriate balancing-side force and moments to the mid-symphyseal symmetry plane, although the time and effort required to calculate those forces and moments accurately might negate any advantages of two-dimensional FEA in terms of modelling time. As a test, we estimated the total force transmitted across the symphysis as the sum of all balancing-side forces acting on the mandible and distributed this force equally across exposed mid-symphyseal nodes (experiment 2b, electronic supplementary material, figure S3). Although results of this simulation show a marginal improvement in similarity of strain patterns laterally, the hemi-mandible model with balancing-side forces still fails to reproduce the strain regime of the anterior corpus and lingual symphysis (figure 7*a–d*). Deformation plots (figure 7*e*,*f*) show slight elevation of the anterior toothrow, in contrast to our constrained model and like our three-dimensional complete model, but the posterior corpus and ramus (again) evert (instead of invert).

It is possible that modifications to our loading regime (for example, by applying the mid-symphyseal moments at the symphysis) may produce a more similar strain distribution. However, in the absence of comparative data (either experimental or three-dimensional FEA), compounding layers of model oversimplification that necessitate complicated boundary conditions makes it difficult (if not impossible) to assess model error.

#### 3.2.3. Experiment 3: symphyseal constraints

**Figure 8.**
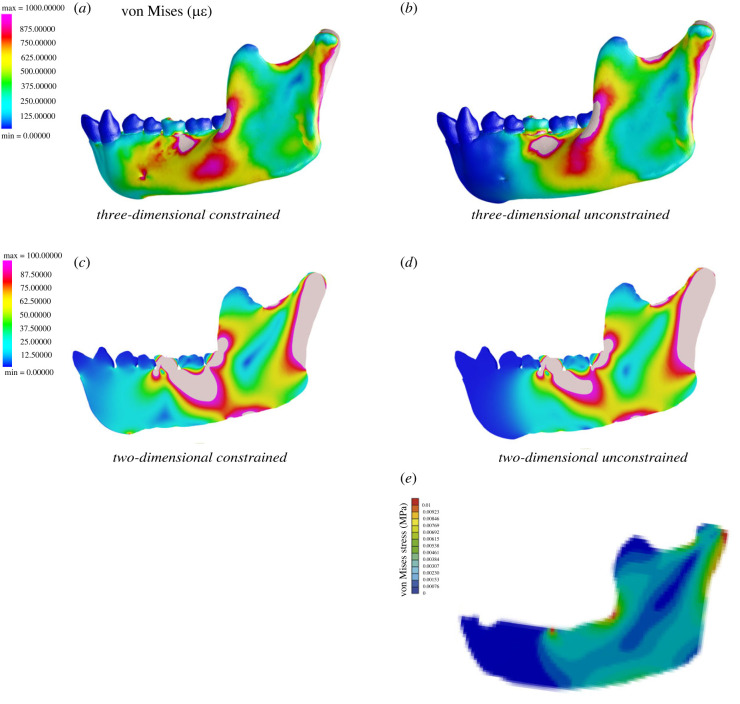
Comparison of all modelling trials (*a*–*d*) with Marcé-Nogué *et al*. [56] (*e*), showing no stress anterior to the bite point. Note that both of our models (*a*) three-dimensional and (*c*) two-dimensional with anterior symphyseal constraints show strains anterior to the bite point while our models that are free at the symphysis (*b*) three-dimensional and (*d*) two-dimensional, do not.

A key difference between the strain regimes of our models (three-dimensional and two-dimensional) and those of Marcé-Nogué *et al*. is the complete absence of strains anterior to the bite point in the latter. Although this is the condition we could expect in an anteriorly unconstrained model, experimental data show that this is not biologically realistic [26,59,61–64]. We hypothesized that removal of symmetry constraints at the symphysis of our models would result in the disappearance of strains anterior to the bite point. Figure 8 illustrates the effect of these anterior restraints on von Mises strain regime as tested in experiment 3 (electronic supplementary material, figure S3). Models in the top row are constrained at the symphysis while models on the bottom row are not. Dark blue regions are unstrained. As predicted, removal of anterior constraints produces an anteriorly truncated strain pattern like the one reported by Marcé-Nogué *et al.* [56].

## 4. Discussion

Researchers who use two-dimensional models to evaluate biomechanical hypotheses about three-dimensional structures purport that such simplified models are quicker and easier to build and yield results that characterize stress and strain patterns in biologically meaningful ways. Indeed, three-dimensional FEA is time consuming, requiring a considerable amount of patience, methodological skill and anatomical knowledge. Therefore, it might be reasonable to consider modelling simplifications as economical trade-offs to reduce time investment and increase rate of research progress if those simplifications were in fact ‘good enough’ at answering the biological/biomechanical questions of interest [81]. But recently published two-dimensional FE studies on primate mandibular biomechanics present results that are inconsistent with those of any published three-dimensional FE studies of the same taxa [26,45,68], including the experimentally validated work of Panagiotopoulou *et al*. (electronic supplementary material, figure S4).

This led us to ask whether a two-dimensional planar model could accurately reproduce the strain and deformation regime of our three-dimensional model of the same specimen. As shown in the modelling experiments presented here, the two-dimensional model fails to recover strain, stress and deformation regimes of the three-dimensional models. Therefore, the evidence does not support the use of two-dimensional models of primate mandibles. Two-dimensional models of the mandible do not contribute meaningfully to our body of knowledge on primate feeding biomechanics.

First, two-dimensional models cannot accurately simulate lateral transverse bending, AP twisting or transverse shear, and hence cannot evaluate their effects on strain regimes. Considering that experimental evidence from bone biology shows that bone is weakest in shear (followed by tension), that bone subject to transverse shear fails in tension [82], and that shear strains influence osteogenesis, particularly when combined with tension [83] (but see also [84]), the complete omission of these metrics is particularly concerning.

Second, two-dimensional models cannot fully model the combination of external forces, and therefore internal loading—shear and moments—acting on the mandible. Primates with differently shaped mandibles also have differently shaped crania, and variation in the angle of the force vector from muscle origin to insertion varies considerably across taxa. Because they are planar, two-dimensional models cannot account for any variation in any degree of mediolateral muscle vector orientation. Our previous work has shown that variation in the size, shape and orientation of masticatory muscles affects mandibular loading and strain regimes [45].

Finally, two-dimensional models cannot accurately reconstruct symphyseal deformation and strain regimes because they do not model the propagation of forces across the symphysis. Although Marcé-Nogué *et al*.'s boundary conditions are unclear, we were able to approximate their strain regime—no strain anterior to the bite point—by removing the anterior constraints from our two-dimensional model. This suggests that the authors treated the anterior-most part of the mandible as the unconstrained terminal end of a planar beam. Not only is this an inaccurate loading regime because it ignores balancing-side forces and moments transferred across the symphysis, it also fails to capture the mechanical effects of realistic symphyseal anatomy. The symphyseal region is an area of particular interest when it comes to comparative biomechanics precisely because it varies so widely in size and shape across taxa. Any comprehensive structural analysis of the mandible should at least consider it.

## 5. Conclusion

Although modelling techniques such as FEA can be powerful tools for understanding form–function relationships in biology, the modelled form (geometry) and resulting function (deformation and strain regimes) must be realistic. Here we show that two-dimensional models of the mandible fail to meet these minimum requirements. Although two-dimensional modelling may appear to be a cost- and time-saving technique, if the results are biologically inaccurate, at best, no time is saved. At worst, the field is misguided. We urge researchers to refrain from drawing functional, ecological or evolutionary conclusions from these models and to validate the results of their models using available experimental data.

## Data Availability

Model data are available from the Dryad Digital Repository: https://dx.doi.org/10.5061/dryad.gmsbcc2r7 [85]. The data are provided in the electronic supplementary material [86].
